# Extending the Functionality of Behavioural Change-Point Analysis with *k*-Means Clustering: A Case Study with the Little Penguin (*Eudyptula minor*)

**DOI:** 10.1371/journal.pone.0122811

**Published:** 2015-04-29

**Authors:** Jingjing Zhang, Kathleen M. O’Reilly, George L. W. Perry, Graeme A. Taylor, Todd E. Dennis

**Affiliations:** 1 School of Biological Sciences, University of Auckland, Auckland, New Zealand; 2 Department of Biology, University of Portland, Portland, Oregon, United States of America; 3 School of Environment, University of Auckland, Auckland, New Zealand; 4 Department of Conservation, Wellington, New Zealand; UC Santa Cruz Department of Ecology and Evolutionary Biology, UNITED STATES

## Abstract

We present a simple framework for classifying mutually exclusive behavioural states within the geospatial lifelines of animals. This method involves use of three sequentially applied statistical procedures: (1) *behavioural change point analysis* to partition movement trajectories into discrete bouts of same-state behaviours, based on abrupt changes in the spatio-temporal autocorrelation structure of movement parameters; (2) *hierarchical multivariate cluster analysis* to determine the number of different behavioural states; and (3) *k-means clustering* to classify inferred bouts of same-state location observations into behavioural modes. We demonstrate application of the method by analysing synthetic trajectories of known ‘artificial behaviours’ comprised of different correlated random walks, as well as real foraging trajectories of little penguins (*Eudyptula minor*) obtained by global-positioning-system telemetry. Our results show that the modelling procedure correctly classified 92.5% of all individual location observations in the synthetic trajectories, demonstrating reasonable ability to successfully discriminate behavioural modes. Most individual little penguins were found to exhibit three unique behavioural states (resting, commuting/active searching, area-restricted foraging), with variation in the timing and locations of observations apparently related to ambient light, bathymetry, and proximity to coastlines and river mouths. Addition of *k*-means clustering extends the utility of behavioural change point analysis, by providing a simple means through which the behaviours inferred for the location observations comprising individual movement trajectories can be objectively classified.

## Introduction

Characterising patterns of behaviour within the movement trajectories of individuals now is a central theme in animal ecology [1–3]. The geospatial lifelines of animals commonly exhibit multiphasic states of behaviour that can be discriminated by distinct geometric combinations of derived movement attributes such as speed and turning angle [4–7]. Methods to infer patterns of behaviour within animal-tracking data sets include state-space models [6,8,9], first passage time [10], maximum entropy [11], positional entropy [12], phase-state models [13], maximum predictive partitioning [14], local fractal dimension [15], Gaussian mixture models [16], partial sum method [17], exponential-segment mixture models [18], and the multi-scale straightness index [19]. Many of these methods are conceptually complex and computational challenging, however, which limits their accessibility to would-be practitioners [6,20–23].

Developed by Gurarie et al. [23], ‘behavioural change point analysis’ (BCPA) is a likelihood-based means of detecting latent structural changes in the parameters underlying locational time-series data. BCPA is performed on components of persistence- and turning-velocity from a continuous-time, Gaussian (Ornstein-Uhlenbeck) process. The method works by sweeping an analytical window over a geospatial lifeline, identifying elements (‘change points’) in the time series where changes in the autocorrelation structure are abrupt. Change points in trajectories are assumed to correspond to discrete shifts in modes of behaviour; many other methods for modelling patterns of behaviour in animal-tracking data are based on this principle [9,12,13,19]. Advantages of BCPA include its robustness to the missing observations and measurement errors that are common in animal-movement data, its computational efficiency and comparative ease of implementation, and its ability to reveal structure in animal-tracking data without prior assumptions regarding the distributions of movement parameters. The method has been applied to several studies of birds and mammals [24–26]. Unlike most other means for inferring changes in behaviour in movement trajectories, however, BCPA does not classify the individual locations comprising animal-tracking data into one of a pre-determined number of mutually exclusive states [27]. This characteristic limits the biological interpretability of individual bouts of behaviour identified by the procedure, and presents challenges for subsequent analysis of BCPA outcomes using standard statistical methods for categorical data (as in [28]).

The most commonly used means of classifying observations or events into discrete groups or categories is *k*-means cluster analysis [29,30]. This method encompasses a number of different fitting algorithms that aim to partition *n* observations into *k* groups, where individual observations are assigned to respective categories (‘clusters’) in such a manner that the degree of association between two observations is maximal if they belong to the cluster and minimal otherwise. Algorithms for *k*-means cluster analysis require that the number of clusters (groups) be specified *a priori*. Comparatively well-known and computationally simple, *k*-means clustering is a simple and effective procedure for grouping bouts of animal behaviour into homogenous same-state classes. Previously, *k*-means clustering has been used to group the individual location observations of animal-movement trajectories into different behavioural modes [31–33]; however, applied in this manner the method neglects useful information about temporal autocorrelation structure that realistically represents behavioural processes.

In this study, we extend the utility of BCPA, by combining it with *k*-means clustering to develop a procedure for classifying the location observations comprising animal-movement trajectories into distinct, mutually exclusive states of behaviour. Our aims are to: (1) evaluate the ability of the method to correctly classify behaviours, using simulated movement trajectories in which the ‘true’ states are known; and (2) demonstrate the operation and utility of the approach, by applying it to high-resolution foraging tracks obtained by GPS telemetry, in a case study of the Little Penguin (*Eudyptula minor*). Our ultimate intention is to describe a simple yet efficient means of exploring the behavioural patterns of free-ranging animals.

## Methods

### Study species and site

The little penguin is the smallest of all sphenisciforms; adults weigh ca. 1 kg. The species is widely distributed along the coastlines of southern Australia, as well as the North, South, Stewart, and Chatham Islands of New Zealand. Primarily nocturnal when at their colonies where they nest close to the shore in burrows, little penguins mainly are found within 25 km of land during the breeding season, but are known to travel farther when not breeding [34]. Adults spend most of the year at sea, but during the breeding season they form pair bonds and alternately undertake short foraging trips that range in length from one to seven days [35]. We chose little penguins as our study species because comparatively little is known about their at-sea behaviour [36,37], their non-threatened conservation status, and the ease with which the study colony could be accessed [37,38].

Our study was conducted in November 2012 on birds from a large colony (>200 breeding pairs) located on Matiu/Somes Island (41.26 S, 174.87 E), a 25- ha pest-free wildlife reserve administered by the New Zealand Department of Conservation that is centrally located in highly urbanised Wellington Harbour. Permission for the study was granted by the New Zealand Department of Conservation (Permit number: WE-34306-FAU); all capture and handling protocols were approved by the University of Auckland's Animal Ethics Committee (AEC/001043).

### GPS telemetry

We used archival GPS data-loggers (‘i-gotU GT120’, Mobile Action Technology) to track the movements of the study animals. Units were waterproofed by sealing them in a single layer of plastic (polyethylene terephthalate, ‘PET’) heat-shrink wrap (0.5 mm wall thickness; DONGGUAN ANPRY PIPE Co. Ltd.). The loggers were ca. 45mm x 25 mm x 8 mm; total weight including the tape used for attachment ranged between 12.3 g and 15.5 g, comprising 1.1% to 1.8% of the weight of the study birds (900 g to 1250 g). GPS receivers were configured to record location estimates at a nominal sampling interval of 1 fix per min, starting at 03:00 local time (UTC+13h) and running continuously thereafter.

### Animal capture and handling

To minimize differences among individuals in body size, reproductive state, and environmental conditions, and to reduce possible deleterious effects on nest attendance, we limited data collection to a 24-h period during the mid-chick-guarding stage. Eight breeding adults were removed from their nest boxes during the afternoon of 6 November 2012, placed in a cloth bag and weighed, then fitted with a GPS data-logger. GPS units were attached dorsally between the flippers with 5–7 strips of 1-cm overlapping waterproof duct tape [39]. After ca. 24 h, individuals were re-captured in their nest boxes and their GPS devices were removed to recover the tracking data.

### Data analysis

**Fig 1:**
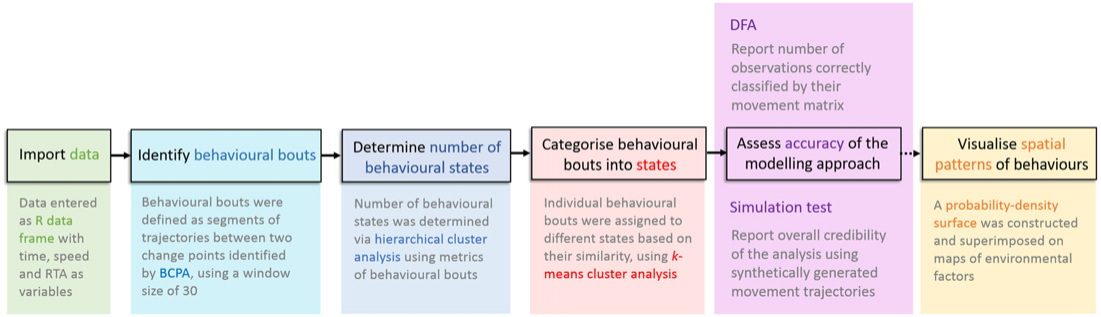
Workflow of analytical methods for inferring and classifying behaviours within animal-movement trajectories using a combination of behavioural-change-point analysis and *k*-means clustering.

The workflow of analytical procedures for inferring and classifying behaviours within the tracking data is described in Fig 1. Location observations obtained from the GPS loggers were processed to calculate speeds and relative turning angles (RTAs) between all sequential pairs of position fixes comprising individual movement trajectories. Code available in the R programming language [40] provided by Gurarie et al. [23] was adopted and modified to execute the BCPA. We used a sub-sampling window size of 30 sequential location observations to meet the minimum sample size required for the Bayesian Information Criterion (BIC) to be used for model selection, and to identify changes in behaviour at the smallest temporal scale possible for BCPA. Segments of trajectories between ‘change points’ identified by BCPA hereon are referred to as ‘bouts’. Because the distributions of speed and the RTA values were strongly positively skewed, we calculated medians from the output metrics (rather than using means), and use them to further classify behaviours; such values subsequently were used as inputs in the *k*-means clustering.

We determined the number of distinct behavioural states for each study animal, through assessment of within-group sums of squares and serial classification of bouts, following the hierarchical cluster method of Kraznowski and Lai [41]. Individual bouts of same-state behaviour were classified into one of three mutually exclusive states based on combinations of median speeds and RTAs, using the *k*-means clustering algorithm of Hartigan and Wong [42] in the statistical software R (version 3.0.1) with the packages ‘cluster’ and ‘fpc’ [43,44]. Thus, bouts identified by BCPA were assigned to unique behavioural states based on similarities of patterns of movement. Proportions of time spent in each behavioural state by the penguins and the numbers of state changes that occurred per hour were then calculated. To determine which of the two movement metrics best differentiated the three behavioural states, we conducted a linear discriminant function analysis (DFA) for each penguin’s trajectory (in Statistica v. 9 [45]) using speed and RTAs of all observed locations as predictor variables. These values were then transformed to standard ‘z’ scores (i.e., the signed number of standard deviations from means), so that the two movement metrics were weighted equally in subsequent analyses. This analysis is reported in terms of the mean variance explained by the predictor variables and the mean number of observations of each behavioural state that were correctly classified by their movement metrics. Unless otherwise stated, all statistical values are reported as means ± standard errors (*SE*).

We assessed how accurately the modelling procedure classified behaviour by generating eight synthetic movement trajectories that represented by multiphasic random correlated walks (in NetLogo v 5.0.4 [46]). Each of these tracks contained 1000 observations, and were comprising three known states of behaviour that were parameterised from the means of empirical distributions of the penguins’ inter-fix speeds and RTAs (S1 Text). BCPA and *k*-means analyses were applied to these trajectories, and the actual states of behaviour at each location were compared to those inferred from the modelling procedure. This approach enabled us to determine the proportions of all observations that were correctly classified.

To visualise spatial variation in patterns of behaviour, we constructed probability-density surfaces of the locations of the state 3 behaviour (foraging) (using the Spatial Analyst extension in ArcMap v. 10.2 [47]), and examined relationships between these surfaces and those of a number of environmental factors (see S2 Text).

## Results

### Simulated tracks

Application of the modelling procedure to the eight synthetic movement trajectories showed that a mean of 93.2 ± 1.2% of State 1 observations, 92.5 ± 0.8% of State 2 observations, and 90.2 ± 0.2% of State 3 observations were correctly classified (overall accuracy: 92.5 ± 0.8%; see S1 Text), indicating that the method performed reasonably well in discriminating the different behavioural modes. On average, there were 197 ± 12.8 behavioural change points per trajectory identified by BCPA. Following *k*-means cluster analysis and thus grouping of the bouts, this number fell to 51.5 ± 4.3 behavioural change points per trajectory.

### Real penguin tracks

**Fig 2:**
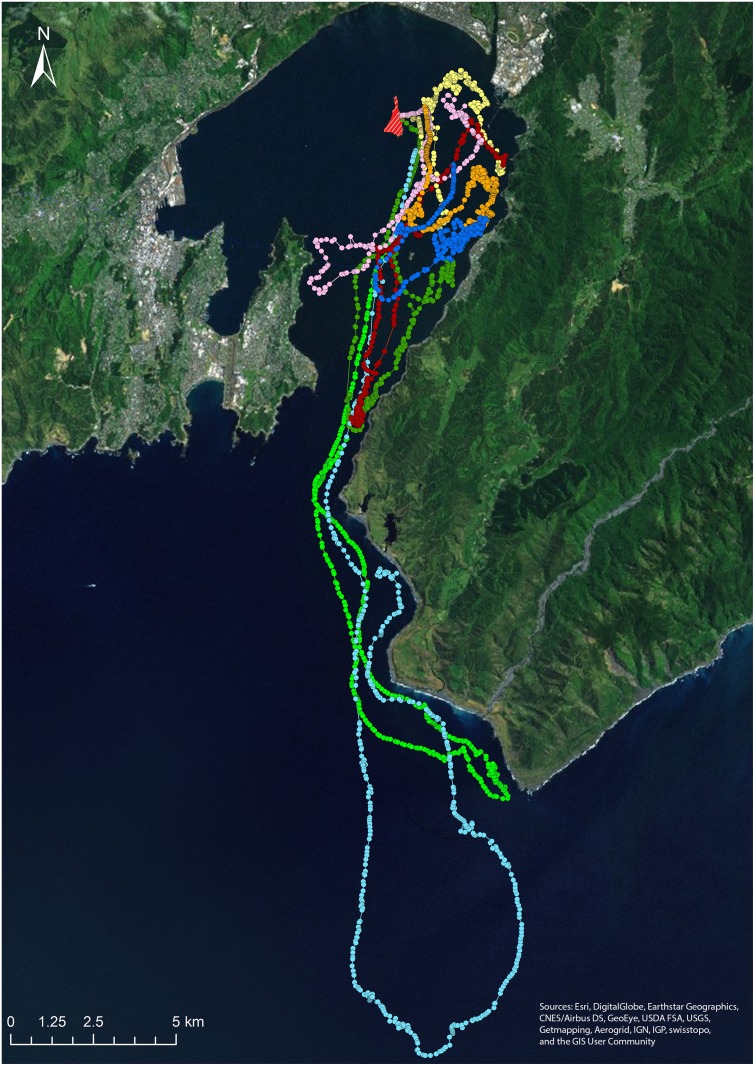
Foraging trajectories of eight little penguins (*Eudyptula minor*) as recorded by GPS data-loggers. The nominal sampling interval of the loggers was 1 fix per min^-1^. Colours represent tracks of different individuals. The location of the island study colony is indicated by red/yellow hatching. Sources of the background satellite images: Esri, DigitalGlobe, Earthstar Geographics, CNES/Airbus DS, GeoEye, USDA FSA, USGS, Getmapping, Aerogrid, IGN, IGP, swisstopo, and the GIS User Community.

Movement trajectories of the eight penguins are shown in Fig 2, and summary characteristics of these data are reported in S1 Table. All trajectories were incomplete in the sense that no bird had returned to its nest box before the batteries of its GPS logger expired, so that the last fixes of all trajectories were recorded when the penguins were away from the colony. Operational periods of the GPS loggers ranged between 9.9 and 13.3 h (mean = 12.0 ± 0.4 h), the numbers of position fixes recorded during foraging trips ranged between 266 and 506 (mean = 398.3 ± 31.6), and the maximum distance from the nest site observed for each bird ranged between 3.5 and 27.4 km (mean = 9.5 ± 3.0 km).

**Fig 3:**
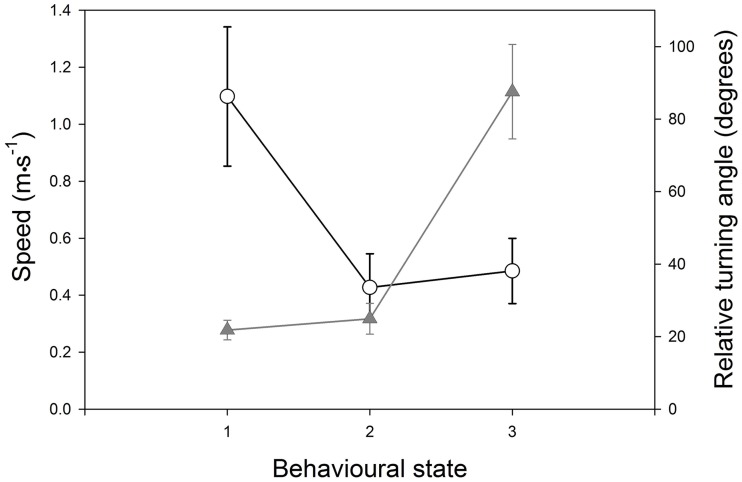
Statistical definitions of behavioural states inferred for the location observations comprising eight penguin foraging trajectories. Behaviours were classified through sequential use of behavioural-change-point and *k*-means cluster analyses, based on combinations of inter-fix speeds (open circles, black lines) and relative turning angles (grey triangles and lines). Circles and triangles represent grand median values of all observations of all penguins, and the vertical bars represent the corresponding inter-quartile ranges.

**Fig 4:**
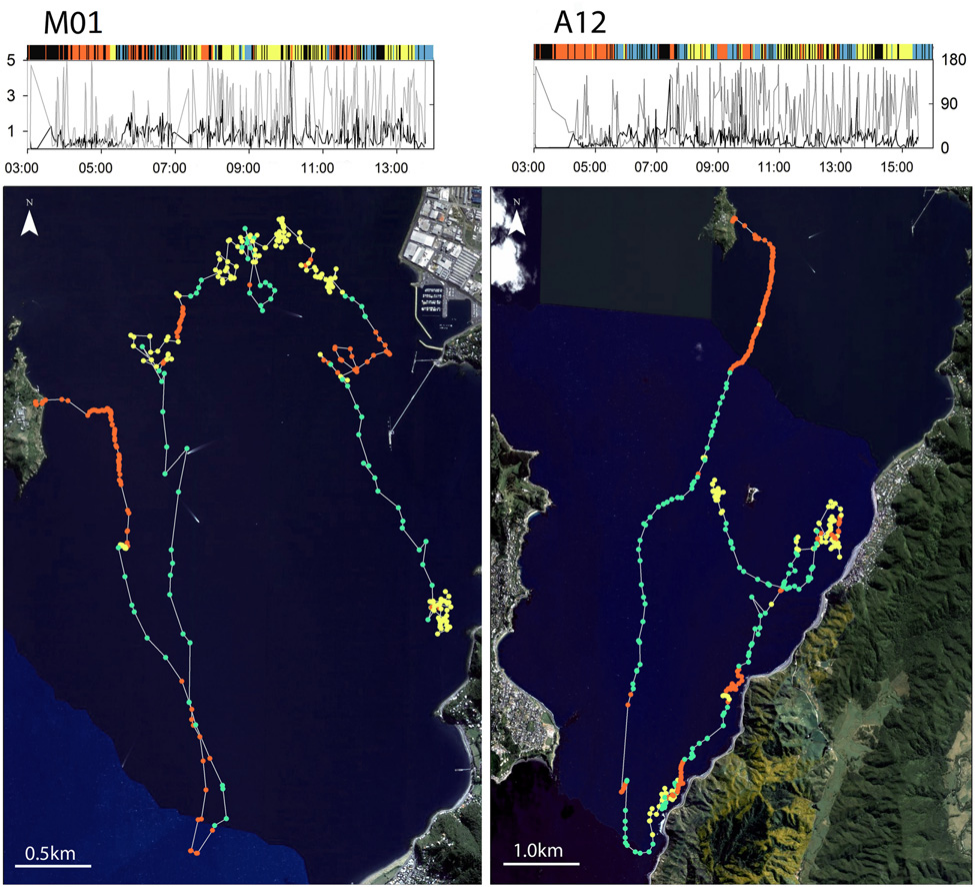
Examples of individual movement trajectories for which GPS-derived location estimates have been classified into discrete behavioural states, penguins A12 and M02. Locations assigned as States 1, 2, and 3 are indicated by blue, red, and yellow points, respectively. Plots above the trajectories show observed inter-fix speeds (ms^-1^; black lines with axis values at the left side of M02 plot) and relative turning angles (degrees; grey lines with axis values at the right side of A12 plot) over time of day. Narrow bars above the plots denote the corresponding inferred states of behaviour.

All of the study penguins exhibited three distinct modes of behaviour in their movement trajectories (Fig 3), except M07, which had only two modes. State 1 was characterised by fast (median speed = 1.1 ms^-1^) and comparatively straight (median RTA = 21.8°) movement trajectories (Fig 4), suggestive of ‘persistent travelling’ or ‘commuting’ behaviour. State 2 was defined by slow swimming speeds (median speed = 0.4 ms^-1^) and moderately low variation in direction (median RTA = 24.9°). This mode of behaviour typically was observed soon after individuals left the colony or immediately following highly tortuous segments of trajectories. Such behaviour can be interpreted as ‘resting’, and appeared to be due to passive displacement on the ocean surface by wind and/or water currents. State 3 was classified by comparatively slow (median speed 0.5 ms^-1^) and highly tortuous (median RTA 87.6°) movements, coupled with frequent gaps of missing locations, indicative of diving behaviour during which the GPS data-loggers were unable to operate. In this state, movements of the penguins were highly localised and restricted in area; such behaviour most likely represents active searching or foraging [37].

Within individual penguin tracks, the proportion of time spent in each of the three behavioural states in most cases were roughly evenly distributed. Excluding penguin M07, State 1 behaviours (travelling/commuting) accounted for between 32.0–43.7% of all observations (mean = 38.5 ± 1.6%), State 2 (resting) between 19.7–38.2% (mean = 30.0 ± 2.1%) of all observations, and State 3 behaviours between 25.3–38.3% (mean = 31.4 ± 2.0%) of all observations. On average, there were 1.1 ± 0.1, 1.0 ± 0.1, 0.9 ± 0.1 bouts per hour of State 1, 2 and 3 behaviours, respectively. The grand mean rate of change of all behaviours was 2.9 ± 0.3 events per hour. The DFA results suggest that speed and RTA accounted for, on average, 75.4 ± 5.4% and 24.6 ± 5.4% of the between-group variability of behavioural-state membership, respectively. The DFA correctly classified, on average, 87.6 ± 9.7%, 92.3 ± 4.5%, and 91.4 ± 5.5% of the behavioural bouts of State 1, 2 and 3, respectively. Overall, 90.5 ± 1.4% of all behaviours were correctly classified.

**Fig 5:**
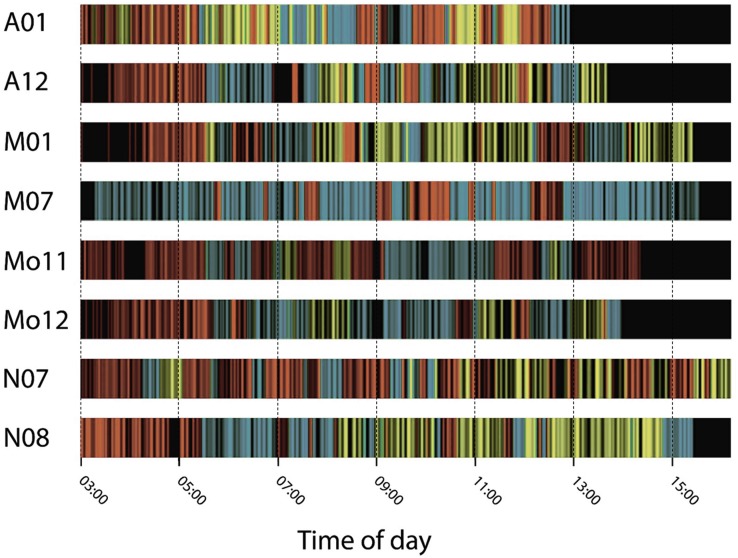
Time series of behavioural states inferred during foraging trips. Colours of vertical bars denote the different states—see Fig 4 for interpretation; black bars denote missing data.

Time series from the combined BCPA/*k*-means clustering procedure demonstrate how the penguins’ patterns of behaviour differed among individuals (Fig 5). Except for bird A01, which initially undertook a sustained period of mixed State 1 and State 2 behaviours, and bird M07, which began its foraging trip in State 1, initial observations of most study animals were classified as State 2 (‘resting’), beginning at ca. 03:00 when the GPS devices first became operational. After several hours, all birds switched from State 2 to State 1 roughly 30 min before sunrise. Following this period, behaviours varied mostly between bouts of State 1 (rapid travelling) and State 3 (‘slow/area-restricted’), suggesting occurrence of a consistent alternating pattern of searching/commuting and intensive foraging.

**Fig 6:**
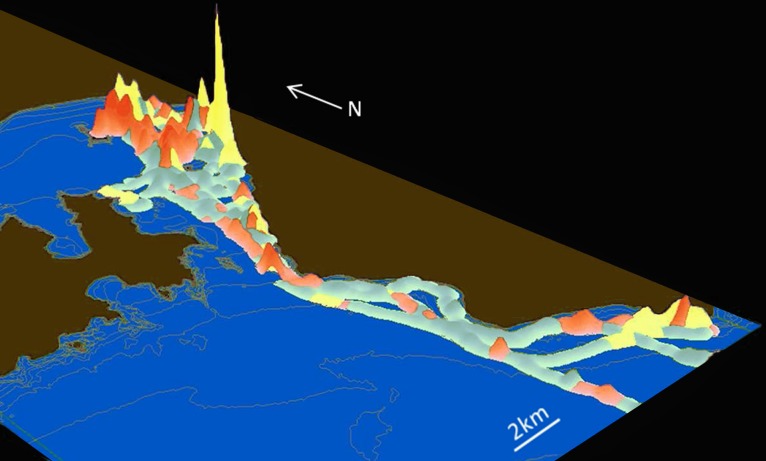
Kernel-density surface of inferred behavioural states superimposed on a map of the study area. Vertical height represents the relative areal density of locations classified as particular behavioural modes. The colours of the behavioural states are the same as those in Figs 3 and 4.

Probability-density surfaces of the locations of inferred states show how patterns of behaviour varied spatially during foraging trips (Fig 6). Compared to State 1 (‘travelling’) and 2 (‘resting’) behaviours, locations classified as State 3 (‘foraging’) were more densely distributed over smaller areas. Locations of State 3 behaviours generally were concentrated in shallow water near the eastern coastline of Wellington Harbour and around river mouths (S5 Fig), suggesting areas where foraging occurred.

## Discussion

Our study demonstrates application of a simple yet effective framework, based on a novel combination of well-known and computationally tractable statistical procedures, for categorising location observations comprising the movement trajectories of animals into discrete modes of behaviour. The method extends the utility of BCPA, by objectively grouping inferred behavioural bouts into standardised classes, thus facilitating interpretation of biological signals. Applied to simulated movement trajectories, the method correctly classified >90% of the ‘true’ modes of behaviour of most individual location observations. Performed on real trajectories of little penguins, the method provided new insights into at-sea behaviour that previously have not been reported.

### Evaluation of the method

The combined BCPA/*k*-means cluster method correctly classified the majority (92.5%) of individual location observations in synthetically generated movement trajectories of known behavioural states (see S1 Text). The few misclassifications of location observations by the BCPA/*k*-means cluster procedure mostly were located at ‘transitional phases’ of bouts (i.e., where behavioural states changed). Similar misclassifications have been reported for other inferential models, and most likely were due to overlapping distributions of movement parameters between the different behavioural modes [48]. Some incorrect bout classifications also may have resulted simply from the difficulties of discretising continuous movements below the resolution of the observational model [6,8].

Benefits of combining BCPA with *k*-means clustering to infer states of behaviour within individual movement trajectories include the broad familiarity of the statistical procedures, as well as their comparative conceptual and computational simplicity. *K*-means cluster analysis is the standard method for grouping objects or events into discrete classes. Open-source code for both analytical procedures is readily available [43,44,49]. Many other inferential models of animal movement require extensive data pre-processing and model training, such as the removal of large outliers, standardisation of intervals between observations, and estimation of the ranges of parameter distributions [28,42]; BCPA is robust to these constraints [23].

### Case study: interpretation of the movement patterns of little penguins

Application of the BCPA/*k*-means clustering procedure provides new insights into the at-sea behaviour of little penguins. The movement patterns of most study birds generally followed a consistent chronological sequence that suggested response to changes in ambient light levels. Before sunrise, most individuals exhibited prolonged bouts of the resting/slow-swimming state, suggestive of a reluctance to move at commuting or active-foraging speeds without being able to visually identify their surroundings, as foraging little penguins rely mostly on visual cues [34,50]. Penguins also use this pre-dawn period for preening and self-maintenance [51]. Finally, the timing of departure from their colony by the penguins may represent evolutionary adaptations to avoid diurnal predators such as skuas (*Stercorarius spp*.) and giant petrels (*Macronectes spp*.) [52]. The abrupt increase in proportion of State 1 observations that occurred for most birds ca. 30 min before dawn is indicative of a shift to sustained commuting behaviour associated with travel to preferred foraging areas [53]. Following this period, behaviour varied more among individuals, as the penguins alternated between area-restricted bouts of foraging and commuting/active-searching bouts of movement to foraging areas, similar to what has been reported by others [53,54]. Patterns of the penguins’ behaviour also appeared to be related to the spatial heterogeneity of their environment. State 3 observations representative of bouts of area-restricted foraging regularly occurred in nutrient-rich shallow waters near coastlines and especially in the vicinity of river mouths, consistent with the foraging patterns of other neritic seabird species [53–55].

## Conclusion

The BCPA/*k*-means clustering method we describe shows promise as a simple exploratory procedure for objectively classifying the individual location observations comprising animal-movement trajectories into discrete modes of behaviour. We recommend further evaluation of the procedure, especially comparison of the accuracy of behavioural-state classification with other available approaches, on both real and simulated animal-tracking data sets.

## Supporting Information

S1 Text Method used to generate synthetic animal-movement trajectories, for comparison between ‘true’ and inferred behavioural states in an example trajectory. (DOCX)

S2 Text Method for construction of kernel-density surfaces of the locations of foraging behaviour in relation to locations of environmental features. (DOCX)

S1 Table Summary characteristics of field deployments of GPS loggers on eight little penguins, 6 November 2012. (DOCX)

S2 Table Parameter values for simulating movement trajectories in NetLogo that were used to test the accuracy of the BCPA/*k*-means cluster modelling procedure. (DOCX)

S3 Table Predictive accuracy of the BCPA/*k*-means cluster procedure for eight synthetic animal-movement trajectories. (DOCX)

S1 Fig Simulated environment and individual movement trajectory used to evaluate the accuracy of the combined behavioural change-point/*k*-means clustering movement model, as implemented in NetLogo v. 5.0.4. Start and end points of the track are indicated by the solid orange circle and open pink square, respectively. The underlying colours of the simulated environment represent ‘resource’ values of the grid that influenced the movement pattern of the model agent. Within the environment, red and blue represent high and low resource values, respectively; in the movement trajectory, colours represent different behavioural states. States 1, 2 and 3 in the trajectory are indicated in blue, orange and yellow, respectively.
(TIF)

S2 Fig Flow chart of the simulation process used to generate synthetic movement trajectories in NetLogo v. 5.0.4. (TIF)

S3 Fig Plot of synthetic trajectory 07 and BCPA outputs in relation to ‘true’ behavioural states. Behavioural states in the trajectory (a) are represented by the same colour scale as that of the ‘true’ behavioural states (b,c): states 1, 2, and 3 indicated by blue, orange and yellow, respectively. The two plots on the right show the time series of velocity and turning angles of the synthetic movement trajectory in (a) decomposed into: (b) turning (Vt = V sin ψ); and (c) persistence components (Vp = V cos ψ). Heavy black lines in the centre of the plots represent running mean values (30-element window), while paired red lines represent one standard deviation. Points in the two plots show running means of individual observations in the time series of Vsin(ψ) and Vcos(ψ); colours reflect the magnitude of temporal autocorrelation, with blues and yellows indicating low and high absolute values (i.e., 0 to 1), respectively. Vertical orange lines indicate change points identified by BCPA. Bar charts in the background of (b) and (c) show the ‘true’ behavioural states of the trajectory at each time step, as defined by the NetLogo model, using the same colour scale as that of the synthetic track.
(TIF)

S4 Fig Comparison between predicted and ‘true’ behavioural states of two methods for inferring behaviour within individual animal-movement trajectories. (a) histograms of speed and relative turning angles of the three ‘true’ behavioural states in the synthetic trajectory. (b) from left to the right: ‘true’ behavioural states as defined by the NetLogo movement model; behavioural bouts identified by BCPA, indicated by different colours; behavioural states inferred by the combination of BCPA and *k*-means cluster analysis; and behavioural states inferred by a switching Markov Chain Monte Carlo model. Start and end points of trajectories and colour scales (except for BCPA output) are the same as indicated in S1 Fig.
(TIF)

S5 Fig Kernel-density surfaces of the locations of foraging behaviour inferred in the movement trajectories of little penguins in relation to water depth and the location of rivers in the study area. Sources of the background satellite image: Esri, DigitalGlobe, Earthstar Geographics, CNES/Airbus DS, GeoEye, USDA FSA, USGS, Getmapping, Aerogrid, IGN, IGP, swisstopo, and the GIS User Community.
(TIF)

S1 Dataset Complete data of all penguin and synthetic movement tracks as presented in the paper. (CSV)
