# Supplementary material for: Extending the Functionality of Behavioural Change-Point Analysis with k-Means Clustering: A Case Study with the Little Penguin (Eudyptula minor)
Source: PLoS One. 2015 Apr 29;10(4):e0122811. doi: 10.1371/journal.pone.0122811 (PMC4414459; doi:10.1371/journal.pone.0122811)
Supplement: S1 Text — (DOCX) [file pone.0122811.s001.docx]

**S1 Text. Method used to generate synthetic animal-movement trajectories, for comparison between ‘true’ and inferred behavioural states in an example trajectory.**

**The model description follows an abbreviated form of the ODD protocol of Grimm *et al.* (**[**1**](#_ENREF_1)**)**.

***Overview***

In this test, we simulated the movements of individuals as they travelling through a spatially correlated landscape. The virtual organisms perceive local habitat quality and make movement decisions as they travelling through patches (as shown in S1 Fig.). For each trajectory that emerges from these individual movements, the behavioural states predicted from modelling approach was compared with the recorded ‘true’ state to assess the predictive accuracy. In this test, the movement decision is a function of the landscape value and structure at a given model time step, thus it serves a mathematical tool to access the validity of BCPA and *k-*means cluster analysis method, without the claim that it represent the true cognitive mechanism of decision making process in animal movement. Simulation of the synthetic tracks was implemented in NetLogo (v 5.0.4) ([2](#_ENREF_2)). Source code for the model is provided at the end of the model description.

**Model environment and model scales** Each model time step represented approximately one minute of real time. The landscape was represented as a 100 × 100 cell lattice (grid), with each cell representing approximately 50 - 100 square meters of land or sea. Grid cells on the lattice were characterised by state variables as set out below.

- *Resource* was the amount of resources of the cell. The resource value was scaled to range between 0 – 1.
- *visit* recorded the number of time agents visited each cell.
- *Habitat* is a categorical parameter, which indicates the resource levels (high, median or low) of each of the grid cells.

**Model entities** Individual animal movements were represented by mobile agents, which changed their behavioural modes according to resource information from the environment (grid cell) that they perceived. The current state of an individual was recorded as the following variables:

- *xcor* recorded the x coordinate of the agent for each model time step;
- *ycor* recorded the y coordinate of the agent for each model time step;
- *state* was one of three possible behavioural states of the agent, which determined the range of parameters of the correlated random walk for the next model step (see details below).

***Design Concepts***

**Landscape structure** We created the landscape with an unconditional Gaussian simulation (in R with package ‘gstat’ ([3](#_ENREF_3))), which is commonly used by spatial modellers to generate correlated random field. This method works by first generating a matrix with values drawn from a standard normal distribution (N ~ (0, 1)). A covariance model was then applied to the matrix raster from a variogram using the parameters specified in S2 Table, so that covariance between values of any two location is a function of distance between the two locations. This means values of nearby locations are stronger correlated than values of locations that are far apart. The resulting raster constituted one unconditional realization. Values from the matrix were then standardized (to the range 0-1) and assigned to each of the grid cells in NetLogo (S2 Table). Details of the unconditional Gaussian simulation method are provided by Dietrich and Newsam ([4](#_ENREF_4)).

**Foraging trips of agent** Foraging behaviour was influenced by landscape value and structure. If an agent entered a higher-resource grid, it would exhibit area-restricted movement defined by lower inter-fix speeds and higher relative turning angles. When an agent exited a high-resource grid, its movements reverted to relatively straight movement. If an agent entered a lower-resource grid, it would display commuting behaviour defined by high inter-fix speeds and low relative turning angles. When an agent exited a low-resource gird, its speed would decrease. If an agent entered a median-resource grid, it would exhibit resting behaviour, which was characterised by low speed and low relative turning angle. Movement ‘emerged’ from the interactions between individuals and the landscape. Movement behavioural state of the agent at the next model step depended on the resource value of the patch the organism was currently located in. The ‘behaviours’ of an individual were driven by landscape structure. Most of the model parameters had stochastic components - some parameters were fixed but represented distributions rather than single point estimates and these were used probabilistically.

***Details***

S2 Fig. describes the sequence of events in each model time step.

**Rules of individual movements** At each time-step, the speed and relative turning angle (RTA) were drawn from the appropriate normal distribution to mimic three distinct types of correlated random walk movements: fast and straight movement (State = 1 travelling/commuting), slow and straight movement (State = 2 resting), and slow and tortuous movement (State = 3 foraging/area restricted behaviour). The three behavioural states had the following parameters:

- If the resource value of the current grid cell was low (between 0.0 – 0.33; 1067 of the possible 10,000 total grid cells), the agent exhibited State 1 behaviour for the next step.
- If the resource value of the current grid was in median range (between 0.33 – 0.67; 7133 grid cells), the agent exhibited State 2 behaviour for the next step.
- If the resource value of the current grid cell was high (between 0.67 – 1, 1260 grid cells), the agent exhibited State 3 behaviour for the next step.

***Model fitting and comparison of results***

For model testing, agent movements were observed, and the model time step, location and 'true' behavioural states of individuals were recorded at each step.

To analyse the synthetic tracks we adopted an identical approach to that applied to the penguin trajectories observed in the field. Eight synthetic tracks were generated from the simulation model, each with 1000 points. We compared the change points identified by BCPA with the changes of recorded ‘true’ states for all tracks. S3 Fig. shows an example synthetic track 07, and break points were identified at abrupt changes of autocorrelation structures by BCPA (indicated as vertical orange lines), which correlated with positions of behavioural changes (represented by background colours). S4 Fig. shows differences between the latent states predicted by the BCPA and k-means clustering analysis (b), and the true states from the same example trajectory (a). We also ran a Hidden Markov model with Bayesian method on this trajectory (c). The data-processing time of the method is minimal: the entire procedure when executed on a data set of 1000 observations required < 5 min on a standard desktop computer; compare this to the several hours needed for a switching Hidden Markov model using a Bayesian filter ([5](#_ENREF_5), [6](#_ENREF_6)) applied to the same data set and run on the same computer, although using a likelihood-based fitting method will require much less time.

Predictive accuracy was calculated by matching the average percentages of model predicted states at each position fix to its ‘true’ states. The average predictive accuracy of the eight synthetic tracks was 92.5 ± 0.8%, and the predictive accuracy values for each behavioural states are listed in S3 Table. We did not consider missing observations or measurement errors that are common observed in GPS data, as their effects on the robustness of BCPA have been discussed in detail in Gurarie *et al.* ([7](#_ENREF_7)).

**NetLogo model code for generating synthetic tracks**

extensions [gis gradient]

globals [color-max

color-min

topography-color]

patches-own [resource

recolor-patch

visit

habitat]

breed [foragers forager]

foragers-own [state

]

to setup

clear-all

set-patch-size 5

resize-world 0 99 0 99

setup-landscape

create-foragers 1

[

set color black

set shape "circle"

set size 2

set xcor 49.5 ; The agent is always released from the centre of the landscape

set ycor 49.5

]

reset-ticks

end

to setup-landscape

let landscape "landscape.txt"

file-open landscape

while [not file-at-end?]

[

let next-x file-read

let next-y file-read

let next-resource file-read

ask patch next-x next-y [set resource next-resource]

]

file-close

set color-max max [resource] of patches

set color-min min [resource] of patches

ask patches

[

set pcolor gradient:scale [ [239 138 98] [247 247 247] [103 169 207] ]

resource color-max 0

; Assign resource values to each grid cell and patch colour are based on these values

if resource > 0.667

[set habitat 3]

if resource <= 0.667 and resource >= 0.333

[set habitat 2]

if resource < 0.333

[set habitat 1]

]

end

to go

ask foragers

[step]

tick

if ticks > 1000 ; Model run 1000 steps

[stop]

end

to step

ask patch-here [

if habitat = 1

[ask foragers[

move1

set state 1

]]

if habitat = 2

[ask foragers[

move2

set state 2

]]

if habitat = 3

[ask foragers[

move3

set state 3

]]

set visit visit + 1

]

pen-down

end

to move1

rt random-normal 0 18

fd random-normal 0.3 0.05

set color green

end

to move2

rt random-normal 0 18

fd random-normal 0.15 0.025

set color red

end

to move3

rt random-normal 0 36

fd random-normal 0.15 0.05

set color yellow

end

**R code for generating landscape**

xy <- expand.grid(1:100, 1:100)

names(xy) <- c('x','y')

a <- 10

while (a <= 100)

{

g.dummy <- gstat(formula=z~1, locations=~x+y, dummy=T, beta=1, model=vgm(psill=0.025,model='Exp',range= a), nmax=20)

yy <- predict(g.dummy, newdata=xy, nsim=1)

References:

1. Grimm V, Berger U, Bastiansen F, Eliassen S, Ginot V, Giske J, et al. A standard protocol for describing individual-based and agent-based models. Ecol Model. 2006;198(1–2):115-26.

2. Wilensky U. NetLogo: Center for connected learning and computer-based modeling. Northwestern University. 1999.

3. Pebesma EJ, Wesseling CG. Gstat: a program for geostatistical modelling, prediction and simulation. Comput Geosci. 1998;24(1):17-31.

4. Dietrich C, Newsam G. A fast and exact method for multidimensional Gaussian stochastic simulations. Water Resour Res. 1993;29(8):2861-9.

5. Morales JM, Haydon DT, Frair J, Holsinger KE, Fryxell JM. Extracting more out of relocation data: building movement models as mixtures of random walks. Ecology. 2004;85(9):2436-45.

6. Postlethwaite CM, Dennis TE. Effects of temporal resolution on an inferential model of animal movement. PLoS ONE. 2013;8(5):e57640.

7. Gurarie E, Andrews RD, Laidre KL. A novel method for identifying behavioural changes in animal movement data. Ecol Lett. 2009;12(5):395-408.
