# Supplementary material for: Extending the Functionality of Behavioural Change-Point Analysis with k-Means Clustering: A Case Study with the Little Penguin (Eudyptula minor)
Source: PLoS One. 2015 Apr 29;10(4):e0122811. doi: 10.1371/journal.pone.0122811 (PMC4414459; doi:10.1371/journal.pone.0122811)
Supplement: S2 Text — (DOCX) [file pone.0122811.s002.docx]

**S2 Text. Method for construction of kernel-density surfaces of the locations of inferred foraging behaviour in relation to locations of environmental features.**

To construct the behavioural density surface, we used The ‘kernel density’ tool in the SURFACE ANALYST module of ArcGIS 10.1 [[1](#_ENREF_1)]. Kernel density distributions were calculated for each penguin’s movement trajectory, and the probability-density surfaces that describe variations in where foraging behaviour occurred were generated. These surfaces were modelled at a spatial resolution of 25m (bandwidth of kernel = 46.7m), using least-squares cross validation [[2](#_ENREF_2)].

We marked areas of several potentially important environmental covariates, including: 1) bathymetry of Wellington Harbour; and 2) the main rivers near Wellington Harbour. These are shown in S5 Fig.

References:

1. ESRI: ArcGIS Desktop: Release 10.1. Redlands, CA: Environmental Systems Research Institute.; 2013.

2. Smyth P: Model selection for probabilistic clustering using cross-validated likelihood. Statistics and Computing 2000, 10(1):63-72.
