# Supplementary material for: Extending the Functionality of Behavioural Change-Point Analysis with k-Means Clustering: A Case Study with the Little Penguin (Eudyptula minor)
Source: PLoS One. 2015 Apr 29;10(4):e0122811. doi: 10.1371/journal.pone.0122811 (PMC4414459; doi:10.1371/journal.pone.0122811)
Supplement: S1 Table — (DOCX) [file pone.0122811.s003.docx]

**S3 Table. Summary characteristics of deployments of GPS loggers on eight penguins, 6 November 2012.**

| Penguin no. | No. of Fixes | Time of First Fix | Time of Last Fix | Trip Duration (h) | Maximum Distance  from Colonly (km) | Total Distance Travelled (km) |
| --- | --- | --- | --- | --- | --- | --- |
| A01 | 483 | 03:02 | 12:58 | 9.93 | 17.13 | 32.93 |
| A12 | 383 | 03:04 | 13:46 | 10.70 | 9.53 | 24.71 |
| M01 | 356 | 03:02 | 15:31 | 12.48 | 3.54 | 20.77 |
| M07 | 506 | 03:19. | 15:39 | 12.50 | 27.36 | 46.12 |
| Mo11 | 266 | 03:02. | 14:26 | 11.40 | 6.04 | 21.37 |
| Mo12 | 287 | 03:02 | 14:03 | 11.02 | 5.78 | 21.84 |
| N07 | 450 | 03:02 | 16:21 | 13.33 | 4.18 | 21.36 |
| N08 | 449 | 03:02 | 15:33 | 12.52 | 4.64 | 24.05 |
| Mean Values (± 1 *SE*) | 398.3±31.6 | 03:04 | 14:47 | 12.01±0.4 | 9.47±3.0 | 26.93±3.1 |
