# Supplementary material for: Extending the Functionality of Behavioural Change-Point Analysis with k-Means Clustering: A Case Study with the Little Penguin (Eudyptula minor)
Source: PLoS One. 2015 Apr 29;10(4):e0122811. doi: 10.1371/journal.pone.0122811 (PMC4414459; doi:10.1371/journal.pone.0122811)
Supplement: S2 Table — (DOCX) [file pone.0122811.s004.docx]

**S4 Table. Parameter values for simulating movement trajectories in NetLogo that were used to test the accuracy of the BCPA/*k*-means cluster modelling procedure.**

| **Parameter/variable** | **Value/range of values** |
| --- | --- |
| **Landscape** |  |
| *Total landscape size* | 100 × 100 cells |
| *Quality value of a patch* | 0.0-1.0 |
| *Landscape spatial autocorrelation variogram model:*  *Type of variogram*  *Sill*  *Range* | Exponential  0.025  10 |
| ***Individual traits*** |  |
| *Speed of movement*  *State 1*  *State 2*  *State 3* | N (0.3, 0.05)  N (0.3, 0.05)  N (0.3, 0.05) |
| *Relative Turning Angle (RTA)*  *State 1*  *State 2*  *State 3* | N (0, 18 ^o^)  N (0, 18 ^o^)  N (0, 36 ^o^) |
| ***Population*** |  |
| *Initial individuals initialised* | 1 per run |
| *Total number of runs* | 8 |
| ***Temporal scale*** |  |
| *Total number of steps* | 1000 |
