# Supplementary material for: Extending the Functionality of Behavioural Change-Point Analysis with k-Means Clustering: A Case Study with the Little Penguin (Eudyptula minor)
Source: PLoS One. 2015 Apr 29;10(4):e0122811. doi: 10.1371/journal.pone.0122811 (PMC4414459; doi:10.1371/journal.pone.0122811)
Supplement: S3 Table — (DOCX) [file pone.0122811.s005.docx]

| Synthetic track no. | Overall accuracy (%) | No. of State 1 obs. | Correctly classified State 1 obs. (%) | No. of State 2 obs. | Correctly classified State 2 obs. (%) | No. of State 3 obs. | Correctly classified State 3 obs. (%) |
| --- | --- | --- | --- | --- | --- | --- | --- |
| 1 | 90.0 | 110 | 90.0 | 846 | 90.1 | 45 | 86.7 |
| 2 | 95.3 | 140 | 95.0 | 684 | 95.3 | 177 | 94.9 |
| 3 | 93.2 | 287 | 89.9 | 534 | 94.1 | 180 | 95.6 |
| 4 | 93.4 | 201 | 93.5 | 654 | 95.1 | 146 | 84.9 |
| 5 | 95.1 | 416 | 95.4 | 262 | 92.4 | 323 | 96.6 |
| 6 | 90.2 | 77 | 88.3 | 653 | 89.3 | 270 | 93.0 |
| 7 | 89.2 | 32 | 96.9 | 714 | 92.9 | 255 | 77.6 |
| 8 | 93.4 | 443 | 96.2 | 519 | 91.1 | 38 | 92.1 |
| Mean Values ± 1 *SE* | 92.5 ± 0.8 | 213.3 ± 54.6 | 93.2 ± 1.2 | 608.3 ± 61.4 | 92.6 ± 0.8 | 179.3 ± 36.3 | 0.90 ± 2.3 |

**S5 Table. Predictive accuracy of the BCPA/*k*-means cluster procedure for eight synthetic animal-movement trajectories.**
