# Supplementary figures and images for: Extending the Functionality of Behavioural Change-Point Analysis with k-Means Clustering: A Case Study with the Little Penguin (Eudyptula minor)
Source: PLoS One. 2015 Apr 29;10(4):e0122811. doi: 10.1371/journal.pone.0122811 (PMC4414459; doi:10.1371/journal.pone.0122811)

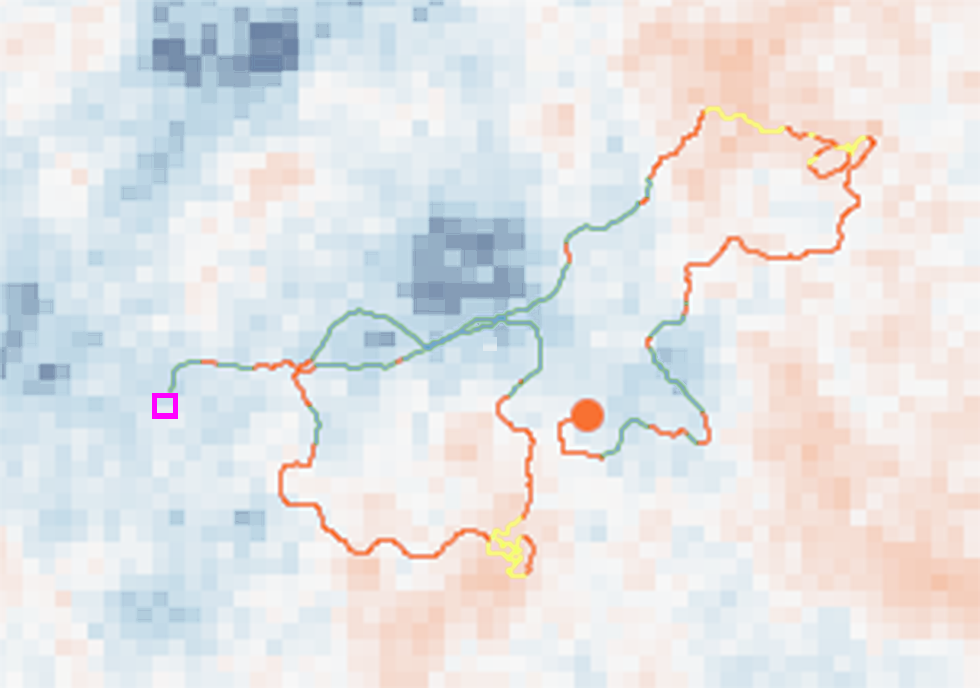

Supplement: S1 Fig — Start and end points of the track are indicated by the solid orange circle and open pink square, respectively. The underlying colours of the simulated environment represent ‘resource’ values of the grid that influenced the movement pattern of the model agent. Within the environment, red and blue represent high and low resource values, respectively; in the movement trajectory, colours represent different behavioural states. States 1, 2 and 3 in the trajectory are indicated in blue, orange and yellow, respectively. (TIF) [file pone.0122811.s006.tif]

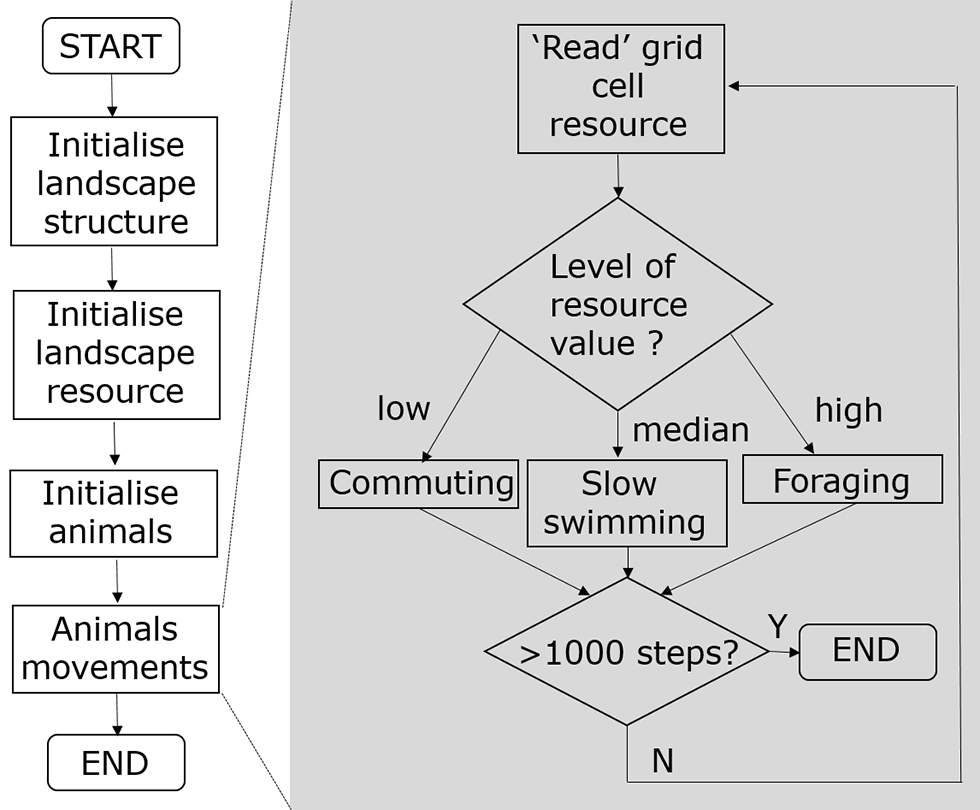

Supplement: S2 Fig — (TIF) [file pone.0122811.s007.tif]

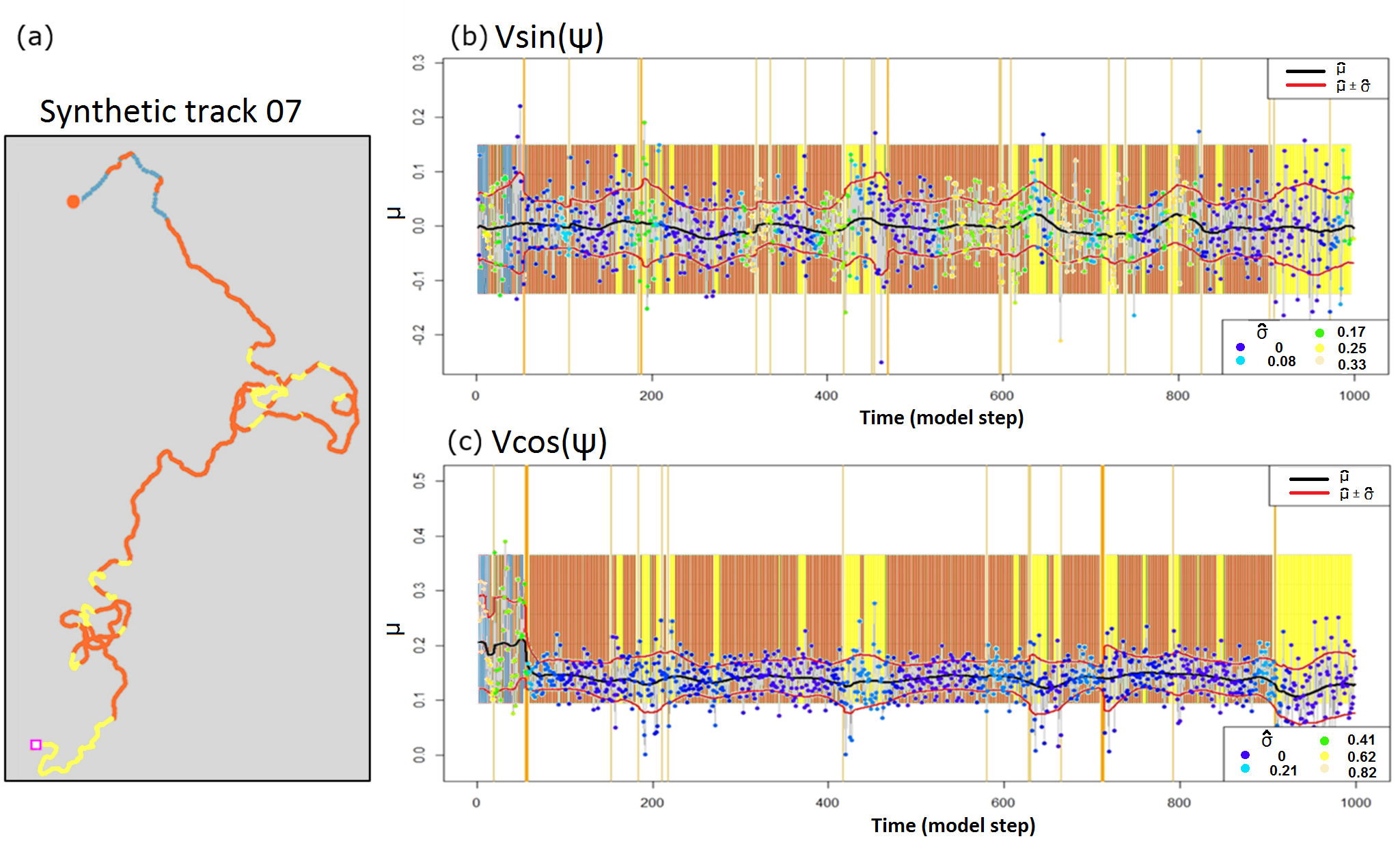

Supplement: S3 Fig — Behavioural states in the trajectory (a) are represented by the same colour scale as that of the ‘true’ behavioural states (b,c): states 1, 2, and 3 indicated by blue, orange and yellow, respectively. The two plots on the right show the time series of velocity and turning angles of the synthetic movement trajectory in (a) decomposed into: (b) turning (Vt = V sin ψ); and (c) persistence components (Vp = V cos ψ). Heavy black lines in the centre of the plots represent running mean values (30-element window), while paired red lines represent one standard deviation. Points in the two plots show running means of individual observations in the time series of Vsin(ψ) and Vcos(ψ); colours reflect the magnitude of temporal autocorrelation, with blues and yellows indicating low and high absolute values (i.e., 0 to 1), respectively. Vertical orange lines indicate change points identified by BCPA. Bar charts in the background of (b) and (c) show the ‘true’ behavioural states of the trajectory at each time step, as defined by the NetLogo model, using the same colour scale as that of the synthetic track. (TIF) [file pone.0122811.s008.tif]

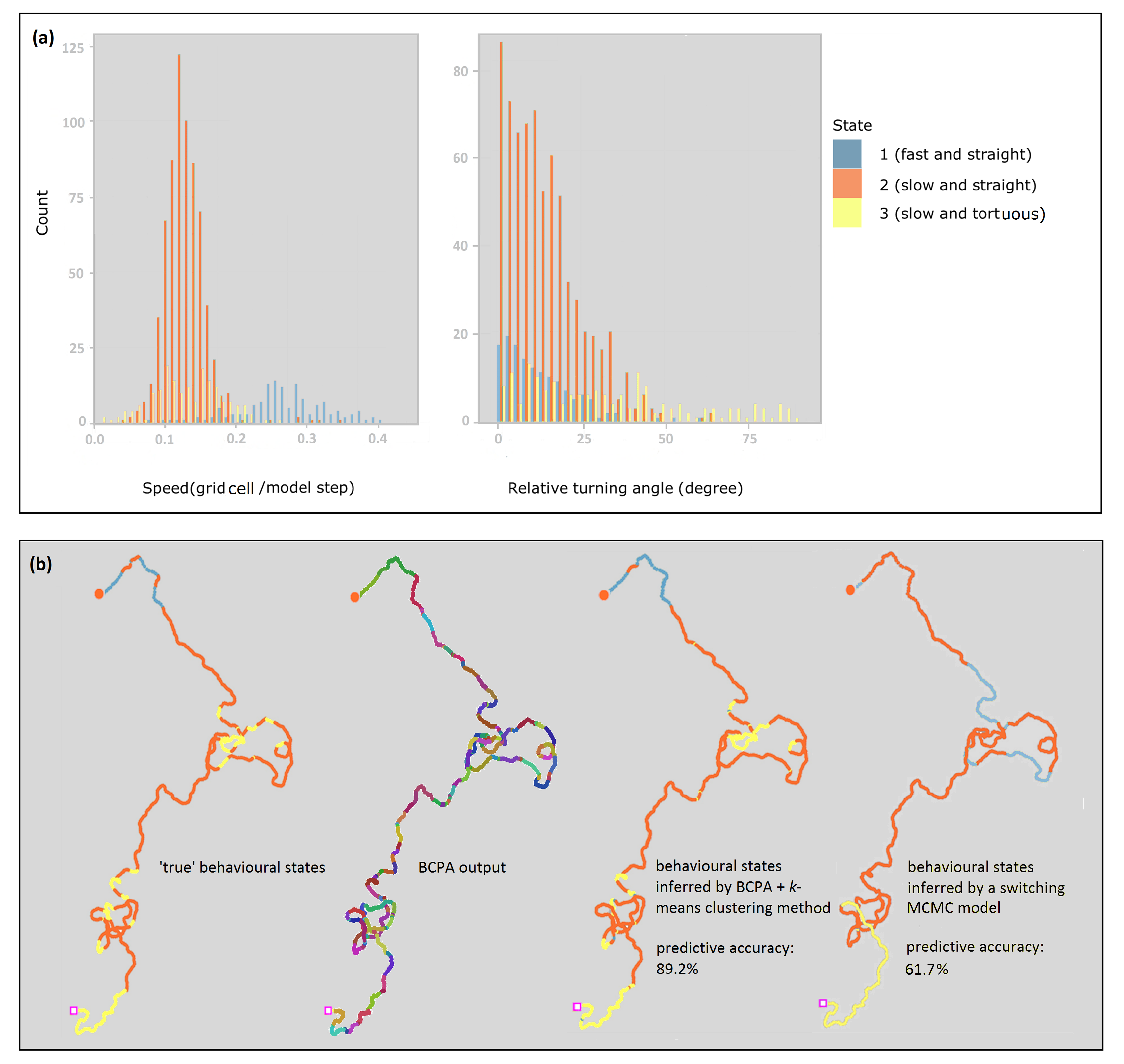

Supplement: S4 Fig — (a) histograms of speed and relative turning angles of the three ‘true’ behavioural states in the synthetic trajectory. (b) from left to the right: ‘true’ behavioural states as defined by the NetLogo movement model; behavioural bouts identified by BCPA, indicated by different colours; behavioural states inferred by the combination of BCPA and k-means cluster analysis; and behavioural states inferred by a switching Markov Chain Monte Carlo model. Start and end points of trajectories and colour scales (except for BCPA output) are the same as indicated in S1 Fig. (TIF) [file pone.0122811.s009.tif]

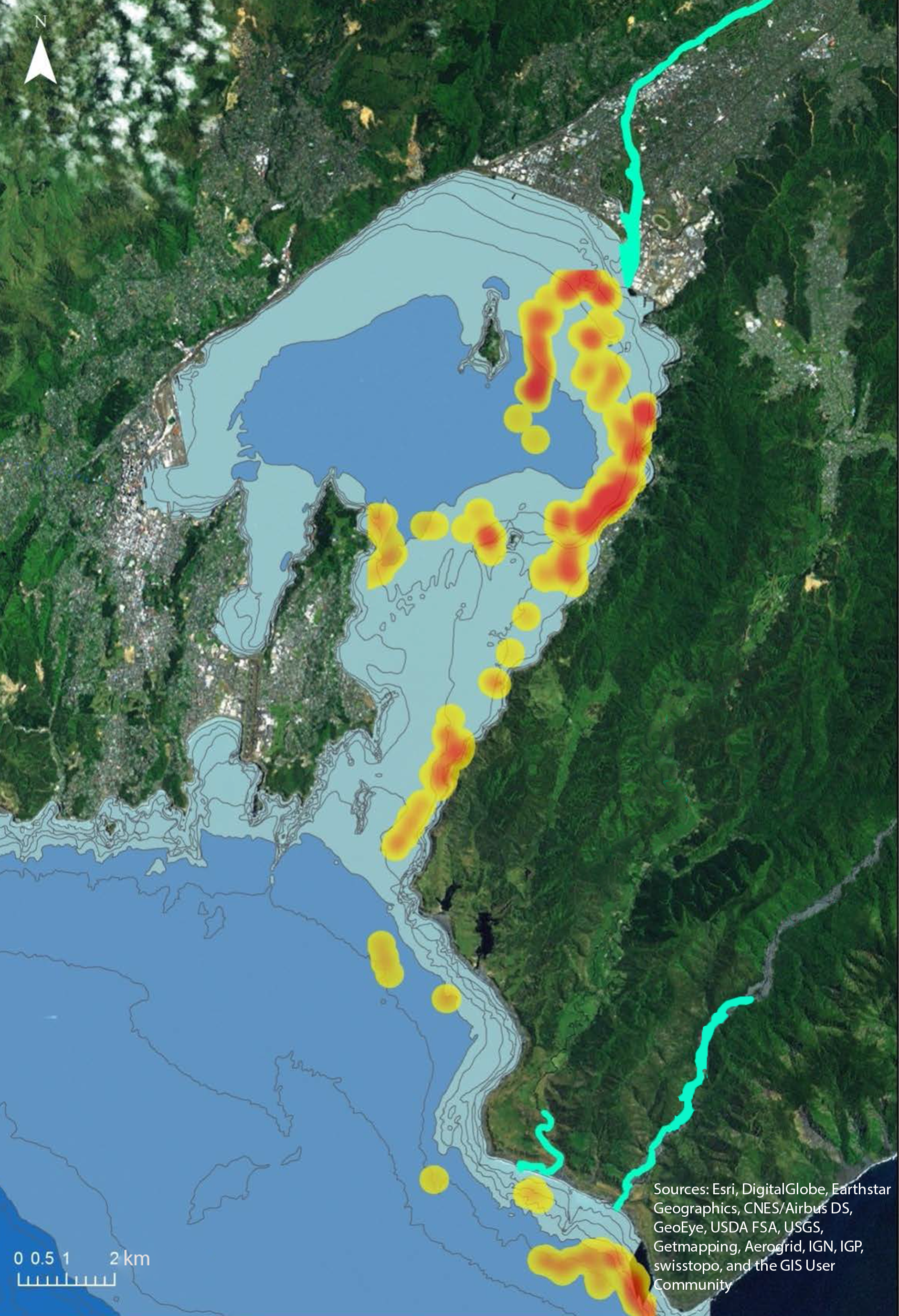

Supplement: S5 Fig — Sources of the background satellite image: Esri, DigitalGlobe, Earthstar Geographics, CNES/Airbus DS, GeoEye, USDA FSA, USGS, Getmapping, Aerogrid, IGN, IGP, swisstopo, and the GIS User Community. (TIF) [file pone.0122811.s010.tif]
